# Comparison between no fasting vs. fasting in patients undergoing cardiac catheterization laboratory procedures: a systematic review and meta-analysis

**DOI:** 10.1093/ehjopen/oeaf123

**Published:** 2026-04-30

**Authors:** Pedro Segura-Saldaña, Mayita Alvarez-Vargas, Victor Ludeña-Meléndez, Juan Llaja-Socorro, Carlos Davila-Paredes, José Sánchez-Cárdenas, Estefani Galvez-Rodriguez, Arón Santillán-Rodríguez, Carlos Cabrera-Cruzado, Pedro Guerra-Canchari, Carlos Diaz-Arocutipa

**Affiliations:** Laboratorio de Ingeniería Biomédica, Departamento de Ingeniería, Facultad de Ciencias e Ingeniería, Universidad Peruana Cayetano Heredia, 430 Honorio Delagado Avenue, San Martin de Porres, Lima 15102, Perú; Unidad de Investigación en Cardiología (UNICAR), Hospital Nacional Edgardo Rebagliati Martins, 490 Edgardo Rebagliati Avenue, Jesus Maria, Lima 15072, Perú; Laboratorio de Ingeniería Biomédica, Departamento de Ingeniería, Facultad de Ciencias e Ingeniería, Universidad Peruana Cayetano Heredia, 430 Honorio Delagado Avenue, San Martin de Porres, Lima 15102, Perú; Unidad de Investigación en Cardiología (UNICAR), Hospital Nacional Edgardo Rebagliati Martins, 490 Edgardo Rebagliati Avenue, Jesus Maria, Lima 15072, Perú; Unidad de Investigación en Cardiología (UNICAR), Hospital Nacional Edgardo Rebagliati Martins, 490 Edgardo Rebagliati Avenue, Jesus Maria, Lima 15072, Perú; Universidad Nacional de Trujillo, Juan Pablo II Avenue, Trujillo 13011, La Libertad, Perú; Sociedad Científica de Estudiantes de Medicina de la Universidad Nacional de Trujillo, 338 Roma Avenue, Trujillo 13011, Peru; Facultad de Medicina, Universidad Nacional de Trujillo, 338 Roma Avenue, Trujillo 13011, Perú; Unidad de Investigación en Cardiología (UNICAR), Hospital Nacional Edgardo Rebagliati Martins, 490 Edgardo Rebagliati Avenue, Jesus Maria, Lima 15072, Perú; Universidad Nacional de Trujillo, Juan Pablo II Avenue, Trujillo 13011, La Libertad, Perú; Facultad de Medicina, Universidad Nacional de Trujillo, 338 Roma Avenue, Trujillo 13011, Perú; Círculo de Extensión Socio-Cultural Daniel Alcides Carrión, Facultad de Medicina, Universidad Nacional de Trujillo, 338 Roma Avenue, Trujillo 13011, Perú; Unidad de Investigación en Cardiología (UNICAR), Hospital Nacional Edgardo Rebagliati Martins, 490 Edgardo Rebagliati Avenue, Jesus Maria, Lima 15072, Perú; Universidad Nacional de Trujillo, Juan Pablo II Avenue, Trujillo 13011, La Libertad, Perú; Sociedad Científica de Estudiantes de Medicina de la Universidad Nacional de Trujillo, 338 Roma Avenue, Trujillo 13011, Peru; Facultad de Medicina, Universidad Nacional de Trujillo, 338 Roma Avenue, Trujillo 13011, Perú; Unidad de Investigación en Cardiología (UNICAR), Hospital Nacional Edgardo Rebagliati Martins, 490 Edgardo Rebagliati Avenue, Jesus Maria, Lima 15072, Perú; Universidad Nacional de Trujillo, Juan Pablo II Avenue, Trujillo 13011, La Libertad, Perú; Sociedad Científica de Estudiantes de Medicina de la Universidad Nacional de Trujillo, 338 Roma Avenue, Trujillo 13011, Peru; Facultad de Medicina, Universidad Nacional de Trujillo, 338 Roma Avenue, Trujillo 13011, Perú; Unidad de Investigación en Cardiología (UNICAR), Hospital Nacional Edgardo Rebagliati Martins, 490 Edgardo Rebagliati Avenue, Jesus Maria, Lima 15072, Perú; Universidad Nacional de Trujillo, Juan Pablo II Avenue, Trujillo 13011, La Libertad, Perú; Sociedad Científica de Estudiantes de Medicina de la Universidad Nacional de Trujillo, 338 Roma Avenue, Trujillo 13011, Peru; Facultad de Medicina, Universidad Nacional de Trujillo, 338 Roma Avenue, Trujillo 13011, Perú; Unidad de Investigación en Cardiología (UNICAR), Hospital Nacional Edgardo Rebagliati Martins, 490 Edgardo Rebagliati Avenue, Jesus Maria, Lima 15072, Perú; Universidad Nacional de Trujillo, Juan Pablo II Avenue, Trujillo 13011, La Libertad, Perú; Sociedad Científica de Estudiantes de Medicina de la Universidad Nacional de Trujillo, 338 Roma Avenue, Trujillo 13011, Peru; Facultad de Medicina, Universidad Nacional de Trujillo, 338 Roma Avenue, Trujillo 13011, Perú; Unidad de Investigación en Cardiología (UNICAR), Hospital Nacional Edgardo Rebagliati Martins, 490 Edgardo Rebagliati Avenue, Jesus Maria, Lima 15072, Perú; Universidad Nacional de Trujillo, Juan Pablo II Avenue, Trujillo 13011, La Libertad, Perú; Sociedad Científica de Estudiantes de Medicina de la Universidad Nacional de Trujillo, 338 Roma Avenue, Trujillo 13011, Peru; Facultad de Medicina, Universidad Nacional de Trujillo, 338 Roma Avenue, Trujillo 13011, Perú; Evisalud - Evidencias en Salud, 1045 Mauel Cipriano Dulanto, Pueblo Libre, Lima 15083, Perú; Unidad de Investigación en Cardiología (UNICAR), Hospital Nacional Edgardo Rebagliati Martins, 490 Edgardo Rebagliati Avenue, Jesus Maria, Lima 15072, Perú; Unidad de Revisiones Sistemáticas y Meta-Análisis (URSIGET), Vicerrectorado de Investigación, Universidad San Ignacio de Loyola, 550 La Fontana Avenue, La Molina, Lima 15023, Perú

**Keywords:** Fasting, Cardiac catheterization, Patient satisfaction, Safety, Systematic review

## Abstract

**Aims:**

Fasting prior to procedures in the cardiac catheterization laboratory is an established indication in clinical practice. However, the evidence supporting this recommendation is uncertain.

**Objectives:**

The aim of this systematic review and meta-analysis was to evaluate the impact of non-fasting vs. fasting before cardiac catheterization.

**Methods and results:**

PubMed, Embase, Scopus, and Web of Science databases were searched until September 2024. Randomized controlled trials comparing both strategies were included. Outcomes were hypoglycaemia, aspiration pneumonia, contrast-induced acute kidney injury (AKI), nausea/vomiting, hypotension, and patient satisfaction. Risk of bias was assessed using the RoB 2.0 tool. All meta-analyses were performed using a random-effects model. Six studies were included (*n* = 2736). The mean age ranged from 62 to 70 years, and 31% were female. There was no significant difference in the risk of hypoglycaemia [risk ratio (RR) 0.82, 95% confidence interval (CI) 0.49–1.38], aspiration pneumonia (RR 1.31, 95% CI 0.28–6.05), nausea/vomiting (RR 0.95, 95% CI 0.58–1.55), contrast-induced AKI (RR 1.87, 95% CI 0.94–3.72) between the non-fasting and fasting groups. In contrast, patients in the non-fasting group had a lower risk of hypotension (RR 0.57, 95% CI 0.38–0.86) and higher satisfaction scores (standardized mean difference −1.53, 95% CI −2.11 to −0.96) compared with the fasting group. The risk of bias was judged as some concerns in three of four studies.

**Conclusion:**

Our results suggest that in patients who underwent cardiac catheterization procedures, non-fasting was a safe strategy and provoked higher satisfaction compared to fasting.

## Graphical Abstract

**Graphical Abstract:**
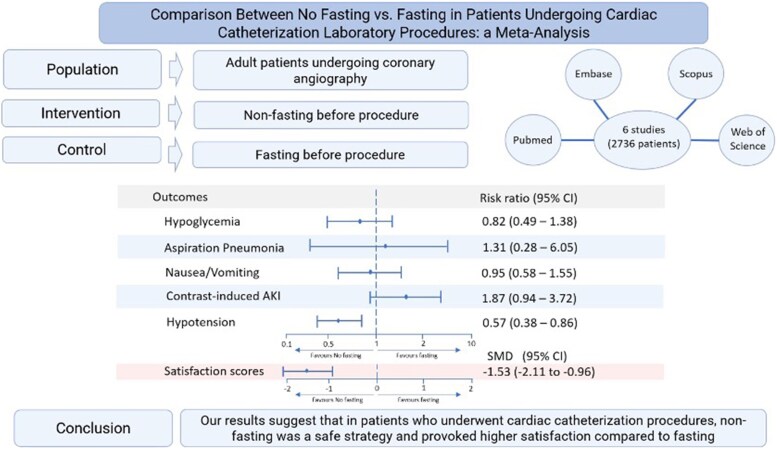

Key Learning Points

- What is already known
  - Fasting prior to cardiac catheterization procedures has been a long-standing clinical practice to reduce risks like aspiration pneumonia.
  - The evidence supporting fasting as a mandatory pre-procedural strategy remains uncertain and limited.
- What this study adds
  - Non-fasting prior to cardiac catheterization is a safe alternative, as it does not increase the risk of hypoglycaemia, aspiration pneumonia, nausea/vomiting, or contrast-induced acute kidney injury.
  - Patients in the non-fasting group experienced a lower risk of hypotension and reported higher satisfaction levels compared to the fasting group.
  - These findings support reconsidering fasting protocols in favour of patient comfort and safety.
  - Larger trials are needed to confirm these findings, especially in specific patient populations such as complex coronary procedures.

## Introduction

Standard preparation for elective cardiac catheterization typically mandates a fasting period of at least 2 h for clear liquids and 6 h of solid foods.^1,2^ Fasting, defined as the controlled abstention from caloric intake for a predetermined duration,^3^ is a common practice aimed at minimizing the risk of aspiration during procedures.^2^ However, extended fasting durations exceeding 12 h may lead to adverse effects such as dehydration, reduced blood volume, hypotension, along with patient discomforts including thirst, hunger, and psychological distress manifested as fatigue, anxiety, and emotional discomfort.^4,5^ Recent studies challenge the necessity of prolonged fasting, suggesting that shorter fasting intervals or even the omission of fasting can reduce complications such as hypotension and hypoglycaemia, while simultaneously enhancing patient satisfaction and maintaining procedural safety.^6–11^ These findings propose that a less restrictive approach to pre-procedural fasting could be both feasible and safe.

This systematic review and meta-analysis aim to evaluate the impact of non-fasting compared to traditional fasting protocols before cardiac catheterization, focusing on metabolic, respiratory, and procedural complications, to inform and potentially refine clinical practice guidelines.

## Methods

This systematic review and Meta-Analyses was reported in accordance with the recommendations of the Preferred Reporting Items for Systematic Reviews and Meta-Analyses (PRISMA) and was registered in the PROSPERO repository (CRD42024588124).

### Search strategy

A comprehensive literature search was conducted in four electronic databases: PubMed, Embase, Scopus and Web of Science. The search terms were ‘fasting’, ‘nothing by mouth’, ‘cardiac catheterization’, ‘percutaneous coronary intervention’, ‘coronary angiography’. The detailed strategy can be found in Supplementary material online, *Table S1*. In addition, the bibliography of retrieved articles was examined for relevant titles. We did not restrict the search by language or date of publication.

### Eligibility criteria

Inclusion criteria were (i) randomized controlled trials; (ii) studies involving adult patients undergoing interventional cardiac procedure such as outpatient or emergency coronary angiography; and (iii) studies reporting information on at least one outcome assessed at any time of follow-up. Review articles, observational studies, and case reports/case series were excluded.

### Outcomes

The outcomes assessed were hypoglycaemia, aspiration pneumonia, contrast-induced acute kidney injury (AKI), nausea/vomiting, hypotension, and satisfaction. The definitions reported in each study were used.

### Study selection

Articles from each database were downloaded to EndNote 20^TM^, and duplicates were removed. Single articles were exported to Rayyan QCRI for screening. Four independent authors reviewed the titles and abstracts of the papers to select potential studies for inclusion. Subsequently, the full-text version of each potential study was independently assessed for eligibility. Any disagreements were discussed with a third author and resolved by consensus.

### Data extraction

The following information was extracted from the included articles: first author, country, study design, selection criteria, sample size, demographic characteristics, comorbidities, fasting and non-fasting group information, use of sedation, cardiac catheterization procedure information, and outcomes.

### Risk of bias assessment

Risk of bias was assessed independently by two reviewers, using the Cochrane Risk of Bias 2.0 (RoB 2) tool for randomized controlled trials. The risk of bias was judged in each domain and overall, as low, some concern, or high risk of bias. Conference abstracts were not included in this assessment due to lack of sufficient information to perform a robust assessment of bias.

### GRADE quality of evidence

The quality of evidence was assessed using the Grading of Recommendations Assessment, Development, and Evaluation (GRADE) approach. This assessment includes the following criteria: risk of bias, inconsistency, indirectness, imprecision, and publication bias. We used the GRADEpro platform (https://gradepro.org/) to elaborate the Table of Summary of Findings.

### Statistical analysis

All meta-analyses were conducted using the inverse-variance method and random-effects models. Paule–Mandel method was used to estimate between-study variance. Effects of non-fasting on outcomes were expressed as relative risks (RR) with their 95% confidence intervals (CIs) for dichotomous outcomes and standardized mean differences (SMD) with their 95% CI for continuous outcomes. Statistical heterogeneity was evaluated using the Cochrane Q test and I² statistic, with values of I² > 50% corresponding to substantial heterogeneity. Publication bias was not assessed because at least 10 studies were not available. Subgroup analysis was also not performed because there was insufficient information for analysis. A sensitivity analysis was performed to evaluate the robustness of the findings by excluding the study by Mishra et al., which was available only as a conference abstract and not peer-reviewed. The aim was to assess whether the inclusion of this study had a significant impact on the pooled effect estimates for all outcomes. The *meta* package in R 4.3.0 (www.r-project.org) was used for all meta-analyses, and a two-tailed *P* < 0.05 was considered statistically significant.

## Results

### Study selection

**Figure 1:**
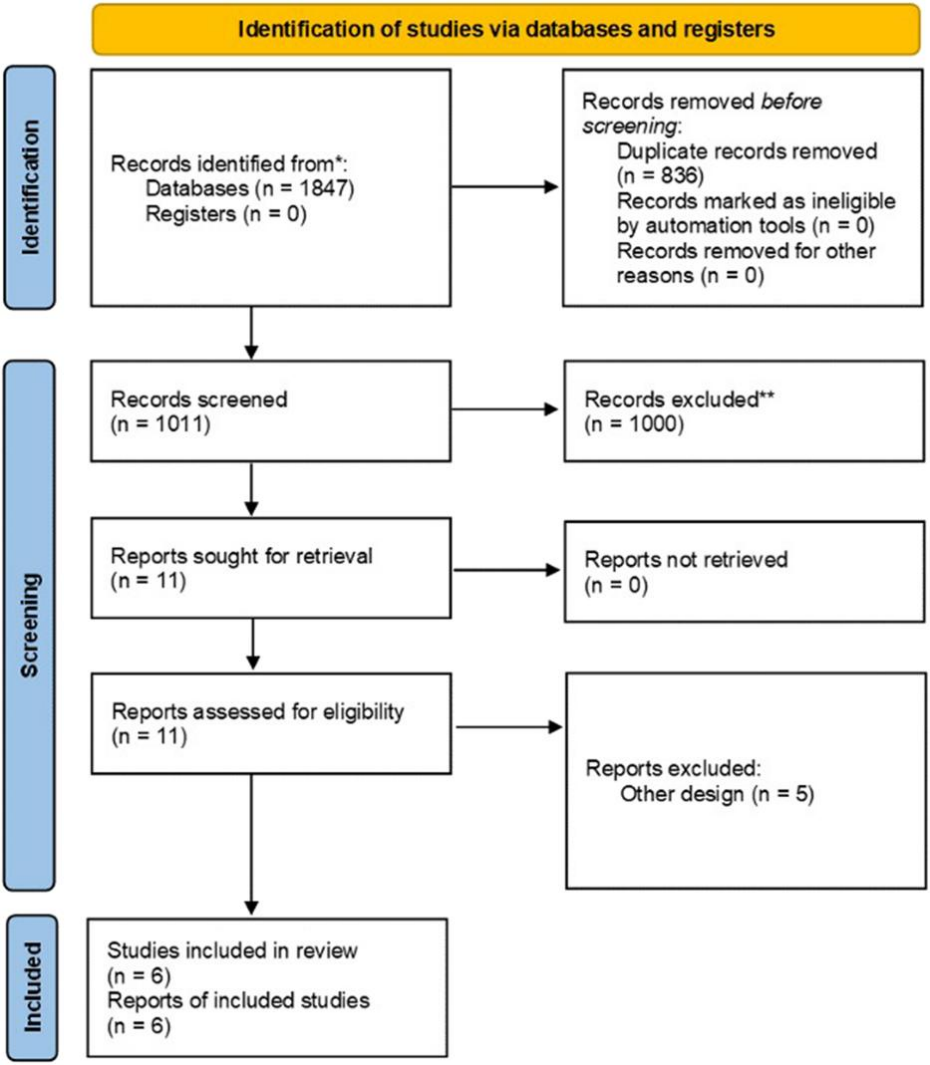
Flow diagram of study selection.

According to our flow chart, 6 studies were selected.^6–11^ Initially, the literature search yielded a total of 1847 studies for initial screening, which were reduced to 11 for the full-text review; of these, 5 were excluded because they were not clinical trials (*Figure 1*).

### Study characteristics

The characteristics of the six included studies are shown in *Table 1*. The studies were conducted between 2017 and 2024. Most studies were conducted in the United States (*n* = 2) or Europe (*n* = 2), and in a single hospital. A total of 2736 patients were included, the mean age ranged from 62 to 70 years. Female participation was generally low, representing 20% to 40% of study populations. The follow-up time of all studies was during hospitalization. the most common comorbidities across studies were hypertension (prevalence between 65% and 79%), diabetes (26–46%), and dyslipidaemia (48–59%). Management protocols for patients in the fasting groups typically required no solid food intake for a minimum of 6 h, with allowance for clear liquids up to 2 h before the procedure. Conversely, non-fasting protocols varied from unrestricted food and drink intake to specific guidelines, such as a light meal consumed up to 2 h before the procedure. The use of pre-procedure sedation was reported in four studies, with two studies using conscious sedation and the rest using discretionary sedation. Procedural characteristics included a predominance of radial artery access, which was used in 87–95% of cases, and varying levels of procedural complexity (*Table 2*). Some studies noted a higher incidence of multi-vessel interventions or specific techniques, such as fractional flow reserve (FFR) assessment or rotational atherectomy. Regarding the type of coronary procedure performed, 60% were diagnostic while the rest were interventional (*Table 2*).

**Table 1:** Characteristics of included studies

Study	Country	Design	Eligibility criteria	Fasting group	No fasting group	Groups	Sample size	Age, years	Female	Comorbidities
Ferreira,^9^ 2024	Australia	Open-label, non-inferiority randomized controlled trial	Age of 18 years and older and referral for coronary angiography, percutaneous coronary intervention, or implantable cardiac device related procedure	Six hours solid food and 2 h clear liquids	No fasting requirement	Fasting	358	70 ± 11.4	35%	Hypertension (70%), diabetes (27%), dyslipidaemia (51%), previous myocardial infarction (20%), previous PCI (19%), previous CABG (6%), heart failure (12%), atrial fibrillation (18%), CKD (22%)
						No fasting	358	69 ± 10.9	34%	Hypertension (70%), diabetes (26%), dyslipidaemia (48%), previous myocardial infarction (25%), previous PCI (21%), previous CABG (8%), heart failure (14%), atrial fibrillation (18%), CKD (19%)
Woods,^6^ 2024	USA	Randomized controlled trial	At least 18 years old, admitted to the inpatient cardiac unit, and scheduled for elective cardiac catheterization with conscious sedation and analgesia	Restricted to nothing by mouth after midnight except for sips of water with medications until the scheduled procedure	Heart-healthy diet group could eat a speciﬁed diet low in fat, cholesterol, sodium, and acidity until the scheduled procedure	Fasting	97	62.7 ± 12.7	38%	-
						No fasting	100			
Tamborrino,^7^ 2024	Italy	Randomized pragmatic trial	All patients admitted to the Cardiology 1 Division with indication for elective coronary angiography	Asked for no solid food and liquid intake for a minimum of 6 and 2 h, respectively, prior to the scheduled procedure	No fasting restriction, and a light meal (two biscuits and 200 mL of tea) allowed up to 2 h prior to catheterization	Fasting	150	69 ± 12	25%	Hypertension (70%), diabetes (35%), dyslipidaemia (56%), obesity (33%), gastrointestinal disease (7%)
						No fasting	150	69 ± 11	33%	Hypertension (67%), diabetes (34%), dyslipidaemia (57%), obesity (31%), gastrointestinal disease (3%)
Boukantar,^8^ 2024	France	Non-inferiority randomized controlled trial	All patients 18 years of age or older who were planned to undergo an elective or semiurgent coronary procedure (angiography and/or PCI)	Fasting for at least 6 h for both solid food and liquids, which is routine practice	Allowed to eat and drink at their convenience (usually with standard meals and drinks provided by the hospital)	Fasting	379	67 ± 12	24%	Hypertension (72%), diabetes (28%), dylipidemia (59%), previous PCI (28%), previous CABG (5%), heart failure (18%), COPD (9%), CKD (16%)
						No fasting	376	68 ± 11	25%	Hypertension (72%), diabetes (30%), dylipidemia (58%), previous PCI (30%), previous CABG (6%), heart failure (15%), COPD (6%), CKD (20%)
Mishra,^10^ 2019	USA	Randomized controlled trial	Subjects who are planned for coronary angiogram or percutaneous intervention, either as outpatients or inpatients	No food after midnight the night before the procedure and will be allowed to drink clear liquids up to 2 h prior to the procedure	No Fasting prior to catheterization. Usual meal on the day of the procedure and allowed to drink as usual	Fasting	131	66 ± 0.99	36%	Hypertension (79%), diabetes (46%), GERD (35%), gastric bypass surgery (0.8%)
						No fasting	122	67 ± 1.16	40%	Hypertension (79%), diabetes (43%), GERD (49%), gastric bypass surgery (0.8%)
Li,^11^ 2017	Singapore	Randomized controlled trial	Patients referred for elective or in-hospital cardiac catheterization	Fasting from midnight as per standard practice	Allowed to eat up to 2 h prior to the planned procedure time	Fasting	266	62.5 ± 11.2	20%	Hypertension (69%), diabetes (42%)
						No fasting	249	62.3 ± 10.7	26%	Hypertension (65%), diabetes (51%)

CABG, coronary artery bypass grafting; CKD, chronic kidney disease; COPD, chronic obstructive pulmonary disease; GERD, gastroesophageal reflux disease; PCI, percutaneous coronary intervention.

**Table 2:** Characteristics of the procedures

Study	Groups	Procedure type	Type of coronary procedure	Complexity of coronary intervention	Vascular access	Contrast volume, mL	Setting	Sedation
Ferreira,^9^ 2024	Fasting	Coronary (83%), device (17%)	Diagnostic (70%), intervention (30%)	Left main artery (2.8%), FFR performed (6.4%), unplanned calcium modification (1.4%)	Radial (94%), femoral (6%)	—	Inpatient (34%), outpatient (66%)	87%, Midazolam: 1.3 ± 0.75 mg, Fentanyl: 40.3 ± 20.4 ug
	No fasting	Coronary (85%), device (15%)	Diagnostic (63%), intervention (37%)	Left main artery (6%), FFR performed (9%), unplanned calcium modification (2.4%)	Radial (94%), femoral (6%)	—	Inpatient (35%), outpatient (65%)	89%, Midazolam: 1.2 ± 0.5 mg, Fentanyl: 41.3 ± 24.4 ug
Woods,^6^ 2024	Fasting	Coronary (100%)	Diagnostic (57%), intervention (43%)	–	–	–	Inpatient (100%)	Conscious sedation (100%)
	No fasting							
Tamborrino,^7^ 2024	Fasting	Coronary (100%)	Diagnostic (58%), intervention (42%)	≥2 treated vessels (16%)	Radial (89%), femoral (11%)	115.5 ± 68.2	Outpatient (100%)	–
	No fasting		Diagnostic (56%), intervention (44%)	≥2 treated vessels (17%)	Radial (87%), femoral (13%)	123.5 ± 77.5		
Boukantar,^8^ 2024	Fasting	Coronary (100%)	Diagnostic (71%), intervention (29%)	Left main artery (12%), chronic total occlusion (12%), FFR performed (28%), rotational atherectomy (0%)	Radial (94%), femoral (6%)	—	Inpatient (31%), outpatient (69%)	Conscious sedation (21%), midazolam (20%), droperidol (0.5%)
	No fasting		Diagnostic (69%), intervention (31%)	Left main artery (7%), chronic total occlusion (10%), FFR performed (29%), rotational atherectomy (6%)	Radial (95%), femoral (5%)	—	Inpatient (35%), outpatient (65%)	
Mishra,^10^ 2019	Fasting	Coronary (100%)	–	–	–	74 ± 4.2	–	–
	No fasting					77 ± 4.7		
Li,^11^ 2017	Fasting	Coronary (100%)	Diagnostic (47%), intervention (53%)	–	–	–	Inpatient (90%), outpatient (10%)	6.8%
	No fasting		Diagnostic (53%), intervention (47%)				Inpatient (39%), outpatient (61%)	2.6%

### Risk of bias assessment

**Figure 2:**
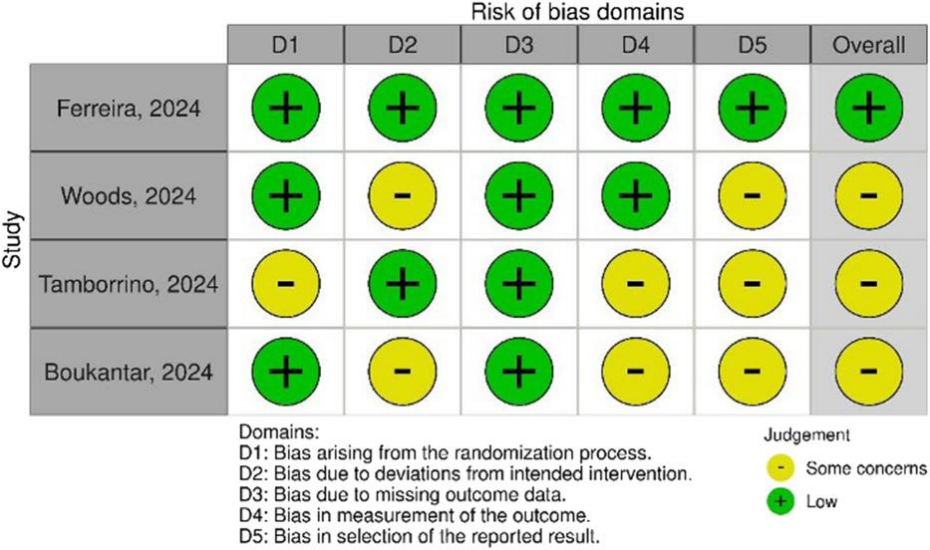
Risk of bias assessment. Central Illustration: Results of the Systematic Review and Meta-Analysis [ADD: unified terminology]: Comparison between no fasting vs. fasting in patients undergoing cardiac catheterization laboratory procedures.

The risk of bias analysis showed that three of the six included studies were at low risk of bias (*Figure 2*). The other three studies had some concerns, especially in domains related to the randomization process, deviations in intervention, and measurement of outcomes.

### GRADE quality of evidence

The quality of evidence was low for all outcomes (*Table 3*). The main reasons for downgrading were imprecision and risk of bias.

**Table 3:** GRADE quality assessment

Outcomes	Anticipated absolute effects* (95% CI)		Relative effect (95% CI)	Number of participants (studies)	Certainty of the evidence (GRADE)
	Risk with Fasting	Risk with Non-fasting			
Hypoglycaemia	31 per 1000	26 per 1000 (15–43)	RR 0.82 (0.49–1.38)	1901 (4 RCTs)^12–15^	⨁⨁◯◯ Low^a,b^
Aspiration pneumonia	1 per 1000	1 per 1000 (0–7)	RR 1.31 (0.28–6.05)	1834 (4 RCTs)^12–15^	⨁⨁◯◯ Low^a,b^
Nausea/vomiting	27 per 1000	26 per 1000 (16–43)	RR 0.95 (0.58–1.55)	2223 (4 RCTs)^12–15^	⨁⨁◯◯ Low^b,c^
Contrast-induced AKI	22 per 1000	41 per 1000 (21–82)	RR 1.87 (0.94–3.72)	938 (3 RCTs)^12,13,15^	⨁⨁◯◯ Low^b,c^
Hypotension	81 per 1000	46 per 1000 (31–69)	RR 0.57 (0.38–0.86)	1484 (3 RCTs)^12–14^	⨁⨁◯◯ Low^d,e^
Satisfaction score	—	SMD 1.53 SD lower (2.11 lower to 0.96 lower)	—	1466 (4 RCTs)^12–15^	⨁⨁◯◯ Low^a,f^

AKI, acute kidney injury; CI, confidence interval; RR, risk ratio; RCTs, randomized controlled trials

^a^Two studies were judged as some concerns.

^b^The sample size does not meet the optimal information size, and the 95% confidence interval was wide.

^c^One study was judged as some concerns.

^d^The risk of bias could not be assessed in two studies.

^e^The sample size does not meet the optimal information size.

^f^The 95% confidence interval was wide.

### Outcomes

**Figure 3:**
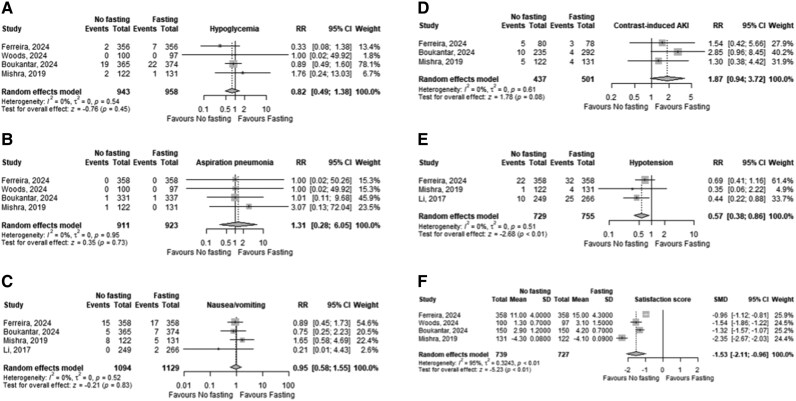
Forest plot of risk ratios for outcomes comparing non-fasting vs. Fasting before cardiac catheterization. Legend: Forest plot showing the effect of no fasting and (*A*) hypoglycaemia, (*B*) aspiration pneumonia, (*C*) nausea/vomiting, (*D*) contrast-induced AKI, (*E*) hypotension, and (*F*) satisfaction score. AKI, acute kidney injury; CI, confidence interval; RR, relative risk; SD, standard deviation.

There was no difference in the risk of hypoglycaemia (RR 0.82, 95% CI 0.49–1.38, *P*-value = 0.45, I^2^ = 0%), aspiration pneumonia (RR 1.31, 95% CI 0.28–6.05, *P*-value = 0.73, I^2^ = 0%), nausea/vomiting (RR 0.95, 95% CI 0.58–1.55, *P*-value = 0.52, I^2^ = 0%), contrast-induced AKI (RR 1.87, 95% CI 0.94–3.72, *P*-value = 0.08, I^2^ = 0%) between the non-fasting and fasting groups (*Figure 3*). In contrast, patients in the non-fasting group had a lower risk of hypotension (RR 0.57, 95% CI 0.38–0.86, *P*-value <0.01, I^2^ = 0%) and higher satisfaction scores (SMD −1.53, 95% CI −2.11 to −0.96, *P*-value <0.01, I^2^ = 95%) compared to the fasting group between the non-fasting and fasting groups (*Figure 3*).

### Sensitivity analysis

When the study by Mishra et al. was excluded from the meta-analysis, the pooled effect estimate remained consistent with the main findings for all outcomes, indicating that the inclusion of this study did not significantly alter the overall results (see Supplementary material online, *Figure S1*).

## Discussion

Our systematic review and meta-analysis of randomized controlled trials demonstrated that non-fasting protocols did not significantly affect the risk of hypoglycaemia, aspiration pneumonia, nausea/vomiting, or contrast-induced AKI when compared to traditional fasting protocols during cardiac catheterization (Central Illustration). However, non-fasting was associated with a lower incidence of hypotension and higher patient satisfaction scores.

Historically, prolonged fasting—6 h for solid food and 2 h for clear liquids—has been standard practice for cardiac catheterization to minimize aspiration risks.^1^ Yet, the benefits of this practice must be weighed against potential drawbacks, such as hypoglycaemia, dehydration, reduced blood volume, and associated hypotension, as well as patient discomfort and psychological stress.^9–12^ Prolonged fasting can mimic a starvation state, inducing insulin resistance, gluconeogenesis, increased lipolysis, and proteolysis.^12,13^

Our systematic review and meta-analysis findings are consistent with previous literature. The absence of significant differences in nausea/vomiting and aspiration pneumonia aligns with the meta-analysis by Choi et al.,^14^ which reported a negligible differential risk of nausea in patients undergoing contrast-based radiological procedures. Similarly, Kwon et al.^15^ found no statistically significant difference in nausea/vomiting incidence between fasting and non-fasting patients undergoing cerebral angiography (odds ratio 0.49, 95% CI 0.17–1.40). These outcomes may be attributed to the use of iso/hypo-osmolar contrast media, known to have lower emetic potential compared to hyperosmolar agents, a trend identified in studies since the 1990s.^16,17^

The lower risk of hypotension observed in the non-fasting group could be related to better hydration status and avoidance of prolonged fasting-induced volume depletion. Factors such as intraoperative delays and perioperative fluid losses may exacerbate dehydration and sustained periprocedural hypotension.^9,18,19^ Moreover, contemporary evidence underscores that patient-centered approaches, like eliminating pre-procedure fasting, may prevent perioperative hypotension and its sequelae.

Regarding AKI, while our results showed no significant difference in the incidence of contrast-induced AKI between fasting and non-fasting groups (*P* = 0.08), it remains crucial to recognize that AKI is multifactorial. The risk factors include patient demographics such as advanced age, hypertension, preexisting renal impairment, and volume of contrast used.^20–22^ The American College of Radiology^23^ and the European Society of Urogenital Radiology^24^ have both noted that pre-procedural fasting does not prevent contrast-induced AKI. Thus, future research should explore the interaction between hydration strategies and contrast-induced renal outcomes more deeply.

Patient satisfaction, an essential measure of procedural success, was significantly higher in the non-fasting group. This finding corroborates studies by Kimpton et al.^25^ and Mitchell et al.,^26^ suggesting that patient well-being improves when fasting is omitted. Beyond comfort, non-fasting may reduce preoperative anxiety, enhance cooperation, and minimize adverse experiences such as dizziness and headache.^27,28^ These benefits suggest that non-fasting protocols could positively influence procedural adherence and outcomes.

While our meta-analysis provides compelling evidence supporting the safety and benefits of non-fasting protocols prior to cardiac catheterization, some limitations should be acknowledged. First, the heterogeneity of the included studies, particularly regarding patient demographics, types of procedures, and fasting/non-fasting protocols, may influence the generalizability of our findings. Second, given that all studies were open-label, there is likely to be a performance bias especially for the outcome of satisfaction. Third, our findings are not necessarily applicable to patients undergoing more complex coronary procedures as they were under-represented in the trials. Further research is warranted in this setting to explore the need for fasting prior to complex procedures that have a longer duration and use more contrast. Fourth, although some of the included trials, such as those by Ferreira et al. and Boukantar et al. were designed as non-inferiority studies, the majority of studies in our meta-analysis followed superiority designs and did not report predefined non-inferiority margins. As such, it was not methodologically appropriate to interpret the pooled estimates using a non-inferiority framework. Additionally, no information was reported on the quantity and quality of food received in the non-fasting group, which could have some impact on the results. Another important limitation is that all included trials were open-label, which introduces a risk of performance and detection bias, particularly for subjective outcomes such as patient satisfaction. Moreover, according to the GRADE framework, the certainty of the evidence remains low due to heterogeneity, imprecision, and the inherent limitations of the included studies. The observed heterogeneity may be partly explained by differences in satisfaction scales, cultural variations in patient expectations, and the use of diverse sedation protocols. Finally, although a non-inferiority approach could have been considered, most included trials were designed as superiority studies and did not define non-inferiority margins; therefore, applying this framework was not methodologically appropriate in our analysis.

Despite these limitations, our findings have significant clinical implications. Shifting towards non-fasting protocols could enhance patient comfort and cooperation, reduce preoperative stress, and decrease the risk of hypotension, which may improve overall procedural outcomes. For clinicians, these results suggest that the rigid application of fasting guidelines before cardiac catheterization should be reconsidered, particularly in elective settings and diagnostic procedures. Importantly, careful patient selection and individualized approaches remain essential, especially for those with high aspiration risk or complex medical histories. Integrating non-fasting protocols into clinical practice could reshape standard preoperative procedures, prioritizing patient-centered care while maintaining safety and efficacy. Beyond patient-centered benefits, our findings also have practical implications for healthcare systems. Eliminating or modifying fasting protocols could streamline pre-procedural logistics, reduce unnecessary cancellations, and improve patient flow in high-volume catheterization laboratories. The substantial heterogeneity observed across studies may reflect differences in patient satisfaction scales, cultural variations in expectations, and variability in sedation protocols. Addressing these issues in future trials through the use of standardized, validated tools for patient-reported outcomes would enhance comparability and strengthen the evidence base.

## Conclusions

In patients who underwent cardiac catheterization procedures, non-fasting was a safe strategy and provoked higher satisfaction compared to fasting. Our findings support reconsideration of the traditional fasting approach before elective cardiac catheterization. Further studies are needed to confirm these benefits and assess long-term outcomes, especially in the setting of complex coronary procedures.

## Lead author biography


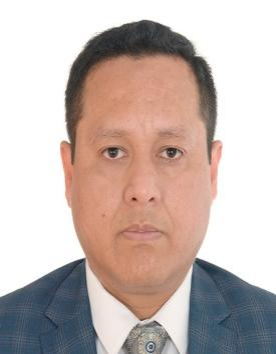


Dr. Pedro A. Segura Saldaña is a Peruvian cardiologist and researcher with advanced training in Health Economics (Pompeu Fabra University) and Biomedical Informatics (UPCH). He is an attending physician at national referral hospitals, professor at Universidad Peruana Cayetano Heredia, and registered researcher with CONCYTEC. Dr. Segura has authored multiple publications in international journals and co-developed patented non-invasive cardiovascular monitoring devices. He has received national awards for clinical innovation and research, and actively participates in ethics committees. His academic focus includes cardiology, digital health, and cost-effectiveness studies in low-resource settings.

## Supplementary Material

oeaf123_Supplementary_Data

## Data Availability

The data underlying this article are available in the article and in its online Supplementary material.
