# Supplementary material for: Comparison between no fasting vs. fasting in patients undergoing cardiac catheterization laboratory procedures: a systematic review and meta-analysis
Source: Eur Heart J Open. 2026 Apr 30;6(2):oeaf123. doi: 10.1093/ehjopen/oeaf123 (PMC13128456; doi:10.1093/ehjopen/oeaf123)
Supplement: oeaf123_Supplementary_Data [file oeaf123_supplementary_data.docx]

**SUPPLEMENTARY DATA**

**Table S1. Search strategy**

| PubMed:  (fasting[mh] OR fast*[tiab] OR “nothing by mouth”[tiab] OR “nil-per-os”[tiab] OR NPO[tiab] OR “nil-by-mouth”[tiab]) AND (“cardiac catheterization”[mh] OR “cardiac catheterization*”[tiab] OR “cardiac catheterization*”[tiab] OR “coronary procedure*”[tiab] OR “percutaneous coronary intervention”[mh] OR “coronary intervention*”[tiab] OR “coronary angiography”[mh] OR “coronary angiography”[tiab]) AND (random*[tiab] OR “clinical trial”[tiab]) |
| --- |
| Embase:  ('fasting'/exp OR fasting OR fast*:ti,ab,kw OR 'nothing by mouth':ti,ab,kw OR 'nil-per-os':ti,ab,kw OR npo:ti,ab,kw OR 'nil-by-mouth':ti,ab,kw) AND ('cardiac catheterization'/exp OR 'cardiac catheterization' OR 'cardiac catheterization*':ti,ab,kw OR 'coronary procedure*':ti,ab,kw OR 'percutaneous coronary intervention'/exp OR 'percutaneous coronary intervention' OR 'coronary intervention*':ti,ab,kw OR 'coronary angiography'/exp OR 'coronary angiography' OR 'coronary angiography':ti,ab,kw) AND (random*:ti,ab,kw OR 'clinical trial':ti,ab,kw) |
| Scopus:  TITLE-ABS-KEY ( ( fast* OR "nothing by mouth" OR "nil-per-os" OR npo OR "nil-by-mouth" ) AND ( "cardiac catheterization*" OR "cardiac catheterization*" OR "coronary procedure*" OR "coronary intervention*" OR "coronary angiography" ) AND ( random* OR "clinical trial" ) ) |
| Web of Science:  TS=( ( fast* OR "nothing by mouth" OR "nil-per-os" OR npo OR "nil-by-mouth" ) AND ( "cardiac catheterization*" OR "cardiac catheterization*" OR "coronary procedure*" OR "coronary intervention*" OR "coronary angiography" ) AND ( random* OR "clinical trial" ) ) |


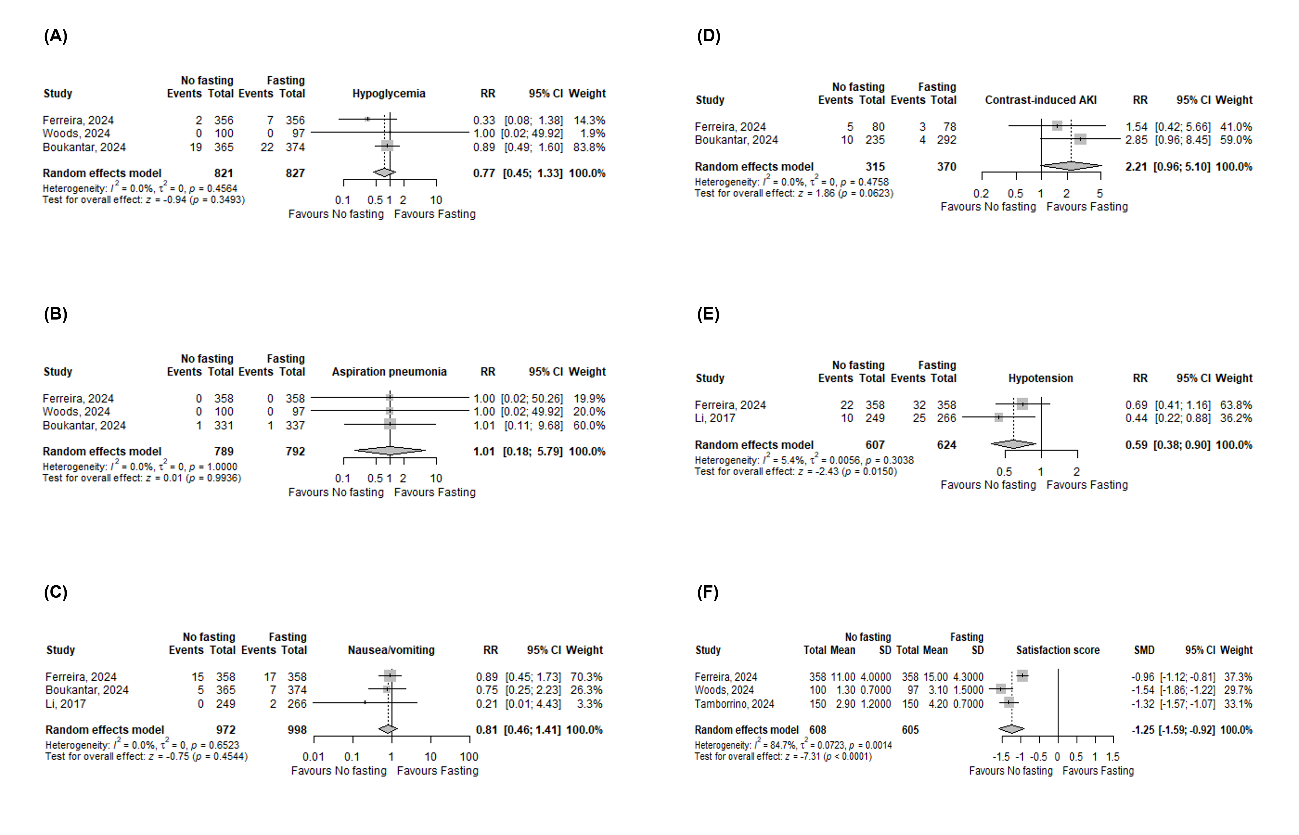


**Figure S1. Forest Plot of risk ratios for outcomes comparing Non-Fasting vs. Fasting before cardiac catheterization, excluding Mishra 2019 et al.**
